# Birth Weight and Its Relationship with the Cardiac Autonomic Balance in Healthy Children

**DOI:** 10.1371/journal.pone.0167328

**Published:** 2017-01-17

**Authors:** Livia Victorino Souza, Vanessa Oliveira, Franciele De Meneck, Ana Paula Grotti Clemente, Maria Wany Louzada Strufaldi, Maria do Carmo Franco

**Affiliations:** 1 Nephrology Division, Federal University of São Paulo, São Paulo, Brazil; 2 Nutrition Department, Federal University of Alagoas, Alagoas, Brazil; 3 Pediatric Department, Federal University of São Paulo, São Paulo, Brazil; University of Southampton, UNITED KINGDOM

## Abstract

Several studies indicate that the fetal environment plays a significant role in the development of cardiometabolic disease later in life. However, a few studies present conflicting data about the correlation between birth weight and the impairment of cardiac autonomic modulation. The purpose of the present study was to provide further knowledge to elucidate this contradictory relationship. One hundred children aged 5 and 14 years had anthropometric parameters, body composition and blood pressure levels determined. Heart rate variability (HRV) was evaluated by heart rate monitoring, including measurements of both the time and frequency domains. The results showed inverse correlation between the HRV parameters with BMI (RMSSD: *P* = 0.047; PNN50: *P* = 0.021; HF: *P* = 0.041), systolic (RMSSD: *P* = 0.023; PNN50: *P* = 0.032) and diastolic (PNN50: *P* = 0.030) blood pressure levels. On the other hand, there were consistent positive correlations between the HRV parameters and birth weight (RMSSD: *P* = 0.001; PNN50: *P* = 0.001; HF: *P* = 0.002). To determine the effect of birth weight on HRV parameters, we perform multivariate linear regression analysis adjusted for potentially confounding factors (prematurity, gender, age, BMI, physical activity index and SBP levels). These findings were preserved even after adjusting for these confounders. Our results suggested that impaired cardiac autonomic modulation characterized by a reduction in the parasympathetic activity occurs in children with low birth weight. One possible interpretation for these data is that a vagal withdrawal, rather than a sympathetic overactivity, could precede the development of hypertension and other cardiometabolic diseases in children with low birth weight. However, long-term studies should be performed to investigate this possibility.

## Introduction

Birth weight has been considered an important measure for assessing fetal growth and is an indicator of neonatal morbidity, survival, and development in children [1–3]. It is clear that the processes arising during fetal development can influence health later in life. In fact, adaptations of the fetus to the inadequate intrauterine environment promote alterations in tissue development and function and can result in increased susceptibility to chronic diseases in adulthood [4, 5].

It is known that the two ends of the birth weight spectrum, low and high birth weight, are associated with an increased risk of cardiovascular events in childhood [6–8] and adult life [9, 10]. Previous studies have demonstrated that those born with low or high birth weight have impairment of the arterial vascular system with modifications in both functional (e.g., endothelium dysfunction, reduction in nitric oxide production) and structural/mechanical (e.g., higher arterial intima-media thickness, elastin synthesis deficiency, increased arterial stiffness) properties [8, 9, 11–14]. However, it should also be considered that the high incidence of cardiovascular disorders could be due to alterations in autonomic control. Exposure to an adverse intrauterine environment during sensitive periods of fetal development produces long-term alterations in the autonomic nervous system (ANS) [15, 16, 17]. An increasing body of evidence suggests that low birth weight is correlated with alterations in autonomic responses [15–20]. Weitz et al. [19] found that individuals born with very low birth weight have lower sympathetic nerve activity under resting conditions. Meanwhile, Jones et al. [20] report autonomic dysfunction in adults with low birth weight, characterized by increased sympathetic activity and reduced parasympathetic activity and baroreflex sensitivity. Although some studies have found evidence of a correlation between low birth weight and autonomic dysfunction, the results remain inconclusive. Thus, the purpose of this study was to conduct a cross-sectional study of children stratified by birth weight quartiles to identify alterations in autonomic function through the analysis of heart rate variability (HRV).

## Materials and Methods

This study was a cross-sectional survey conducted in a representative group of children identified in the Youth Healthcare Centre located near the Federal University of São Paulo (UNIFESP, São Paulo, SP, Brazil). A total of 100 children were identified during the study period. No child had any clinical signs of endocrine, renal or cardiovascular disease. Personal and family medical histories were obtained using a questionnaire completed during an interview with the parents. The prenatal data from the mother’s recall was confirmed with medical records. The Ethics Committee of the Federal University of São Paulo approved the study protocol (Number: 214.956). All parents and children signed written informed consent/assent forms. All children were evaluated twice on different days. On the first day, the Baecke questionnaire was administered and body composition was assessed. On the second day, anthropometric measures, blood pressure, and HRV were evaluated. All procedures were conducted in a quiet room with minimum disturbance between 9:00 and 11:00 am. All participants sat still for at least 5 minutes to assess blood pressure and heart rate at rest. All measurements were made by the same trained researcher who was blinded to the clinical data.

### Physical Activity Level

The physical activity level was evaluated by the total score obtained with the Habitual Physical Activity Questionnaire proposed by Baecke et al. [21] and validated in a young adolescent population [22].

### Anthropometry

Body weight and height were measured in light clothing without shoes using a standard balance beam scale. Waist and hip circumferences were measured using inextensible tape. Waist circumference (WC) was recorded at a level midway between the lower rib margin and the iliac crest at the end of normal expiration. Hip circumference (HP) was measured at the maximum circumference of the buttocks. For both measurements, the tape was positioned at a level parallel to the floor, and the waist-to-hip ratio was calculated. Body mass index (BMI) was computed using the formula weight (kg)/height (m2). Children were classified as overweight (+1 SDS) or obese (+2 SDS) according to BMI-for-age following the cut-off parameters of the WHO growth standards [23].

### Body Composition

Body composition was evaluated using bioelectrical impedance (BIA) with a single-frequency hand-to-foot (ImpediMed DF50 monitor, Impedimed, Australia) [24]. Children were asked to avoid physical activity and to consume liquids 2 hours before evaluation to prevent errors in measurement due to fluid imbalances. This evaluation was performed after 5 minutes of rest in the supine position, and the electrodes were applied to the hand, wrist, ankle and foot of the right-hand side of the body. Body fat (%BF) was calculated from BIA data with prediction equations [25].

### Hemodynamic Variables and Heart Rate Variability (HRV)

Blood pressure (BP) levels were measured twice in the right arm with the child in a seated position using an automated oscillometric device (Omron HEM907XL; Omron Healthcare; USA) with an appropriate cuff size. The blood pressure value was the average of three measurements made at 2-min intervals [26]. After this procedure, children were asked to lay silently in a supine position on a bed with minimal body movement and to maintain spontaneous breathing for 10 minutes to record both resting heart rate and short-term HRV using a Polar S810i monitor (Polar Electro OY, Finland; sampling rate = 100Hz) [27, 28]. The same researcher blinded to the clinical data conducted the assessment of all children. To minimize the effect of stress during HRV assessment, we conducted a pilot project two months prior to begin the current study.

### Processing of the HRV Data

The HRV indices were calculated as previously described [27, 28]. Briefly, normal beat to beat intervals (RR) were transferred from Polar S810i to Precision Performance Software by an infrared interface device. These data were processed in a specific previous routine designed in MatLab (Math Works, 6.0 version, USA) for the automatic selection of 5 minutes RR with the smallest variance [29]. Afterwards, these time series lasting 5 minutes were analyzed in Kubios software (Biosignal Analysis and Medical Image Group, University of Kuopio, Finland). In this software, the artifacts were corrected with a moderated filter, and the time-domain components were calculated. The following time-domain indices were analyzed: heart rate (HR), MNN (average of all normal RR interval), SDNN (standard deviation of RR intervals), RMSSD (root mean square of successive differences RR intervals), and pNN50 (percent RR intervals with a difference in duration higher than 50 ms). The frequency-domain components were also evaluated by a 2 power spectrum. The classical frequency low frequency (LF = 0.041–0.15 Hz) and high frequency (HF = 0.15–0.40 Hz) bands were expressed in normalized units (n.u.). Moreover, the ratio of LF/HF was also calculated. Spectral analysis was estimated as recommended using the non-parametric method of fast Fourier Transform Algorithms [28, 29].

### Statistical Analysis

Statistical analyses were conducted using SPSS version 21.0 for Windows (IBM Corporation, USA). All continuous variables were examined for normality with the Shapiro-Wilk test and expressed as the mean ± Standard Deviation (SD). Categorical variables were expressed as percentage. Pearson’s correlation and multiple linear regression analyses were performed to verify the relationship between HRV parameters and independent factors. Multicollinearity was evaluated using variance inflation factor (VIF) and significance level was set at *P*<0.05.

## Results

The present study was conducted on 100 children (63 boys and 37 girls) with a mean age of 8.85 years (Range: 6–14; SD = 1.82 years). The mean BW was 3008 g (Range: 1400 to 4590; SD = 605 g) with birth length 47.1 cm (Range: 39.0–53.0; SD = 3.28 cm). For entire cohort, we found a positive correlation between BW and birth length (*r* = 0.603, *P<* 0.001). Preterm delivery occurred in ten pregnancies. We also found that 33% of children have excess weight and 16% of them were already obese. General characteristics of the children are listed in Table 1.

**Table 1:** General Characteristics and Linear Index Values of Heart Rate Variability (Time and Frequency Domain) in the Study Population (n = 100).

General Characteristics	
Birth Weight (g)	3008 ± 605
Birth Length (cm)	47.3 ± 3.29
Prematurity (%)	
Yes	10
No	90
Age (Years)	8.85 ± 1.82
Gender (%)	
Female	37
Male	63
Weight (kg)	34.83 ± 13.52
Height (cm)	135.83 ± 11.93
BMI (kg/m^2^)	18.59 ± 4.60
WC (cm)	60.55 ± 9.55
HC (cm)	71.99 ± 12.43
BF (%)	25.78 ± 6.77
Nutritional Status (%)	
Normal	67
Overweight	17
Obesity	16
Physical Activity Index	8.18 ± 1.14
SBP (mmHg)	100 ± 7.50
DBP (mmHg)	66 ± 4.20
**Time Domain**	
HR (bpm)	83.89 ± 8.12
MNN (ms)	708.65 ± 85.60
SDNN (ms)	48.98 ± 20.00
RMSSD (ms)	51.45 ±20.64
PNN50 (%)	39.88 ± 12.95
**Frequency Domain**	
LF (n.u.)	52.50 ± 14.25
HF (n.u.)	45.02 ± 13.32
LF/HF Ratio	1.37 ± 0.87

Values expressed as percentage or mean ± SD. BMI—Body Mass Index; WC—Waist Circumference; HP—Hip Circumference; BF—Body Fat; SBP—Systolic Blood Pressure; DBP—Diastolic Blood; HR—Heart Rate; SDNN—Standard Deviation Normal-to-Normal Intervals; RMSSD—Root Mean Square of Successive Differences; pNN50—Percentage of Differences Between Adjacent Normal-to-Normal Intervals that is Greater than 50 Milliseconds; LF (n.u.)—Low Frequency in Normalized Units; HF (n.u.)—High Frequency in Normalized Units.

Considering the HRV analysis, there was no significant gender effects with respect to the time-domain (HR: *P* = 0.098; MNN: *P* = 0.271; SDNN: *P* = 0.208; RMSSD: *P* = 0.624; PNN50: *P* = 0.882) or frequency-domain (LF: *P* = 0.294; HF: *P* = 0.844; ratio LF/HF: *P* = 0.546) indices; therefore, analyses were performed considering boys and girls together. For the entire cohort, inverse correlations were observed between the HRV parameters with BMI (RMSSD: *r* = - 0.199, *P* = 0.047; PNN50: *r* = - 0.231, *P* = 0.021; HF: *r* = - 0.205, *P* = 0.041), systolic (RMSSD: *r* = - 0.227, *P* = 0.023; PNN50: *r* = - 0.214, *P* = 0.032) and diastolic (PNN50: *r* = - 0.217, *P* = 0.030) BP levels. On the other hand, resting HR values were positive correlated with BMI (*r* = 0.300, *P* = 0.002) and blood pressure levels (SBP: *r* = 0.382, *P<* 0.001; DBP: *r* = 0.317, *P* = 0.001).

**Fig 1:**
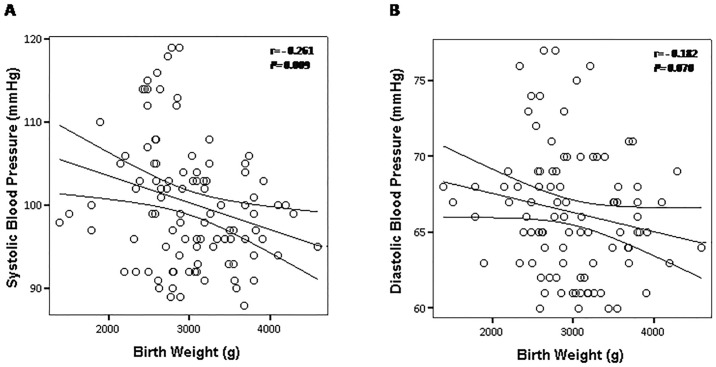
Scatter plots showing the correlation between birth weight with systolic (A) and diastolic (B) blood pressure levels. The lines represent the weighted regression with its 95% confidence interval.

**Fig 2:**
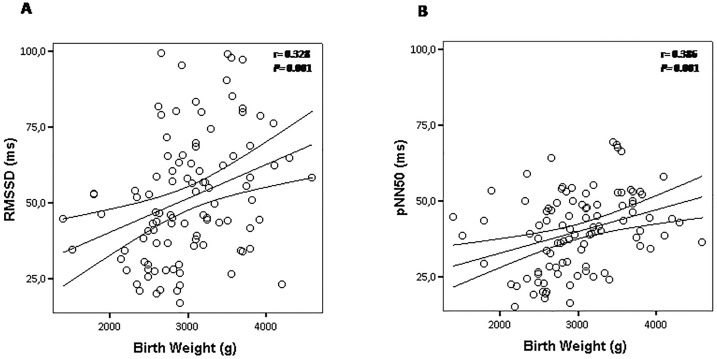
Scatter plots showing the correlation between birth weight with parameters of the time domain: (A) RMSSD and (B) PNN50. The lines represent the weighted regression with its 95% confidence interval.

**Fig 3:**
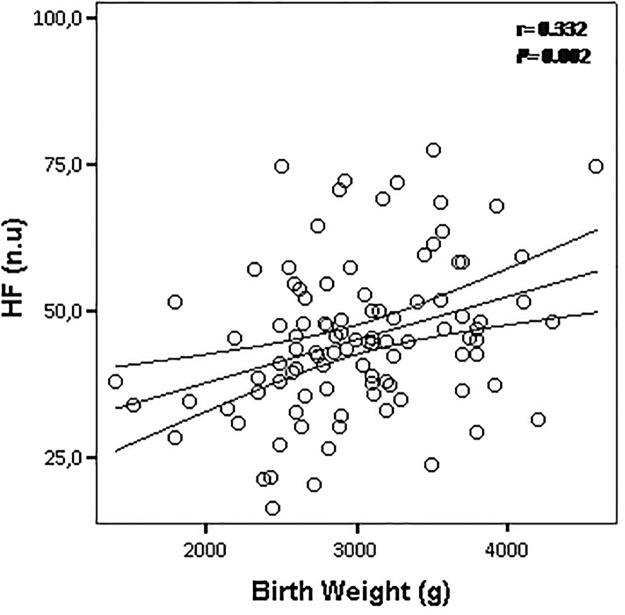
Scatter plots showing the correlation between birth weight with HF, parameter of the frequency domain. The lines represent the weighted regression with its 95% confidence interval.

Additionally, we observed an inverse correlation between BW and systolic BP levels (*r* = - 0.261, *P* = 0.009) (Fig 1A). Meanwhile, diastolic BP (*r* = - 0.182; *P* = 0.070) was not influenced by BW in these children (Fig 1B). Interestingly, there were consistent positive correlations between BW with parameters of the time domain (RMSSD: r = 0.328, P = 0.001; PNN50: r = 0.386, P = 0.001) (Fig 2) and frequency domain (HF: r = 0.332, P = 0.002) (Fig 3), suggesting that impaired vagal modulation to the heart in children is influenced by low birth weight. To determine the effect of birth weight on HRV parameters, we perform multivariate linear regression analysis adjusted for potentially confounding factors (prematurity, gender, age, BMI, physical activity index and SBP levels). When RMSSD was tested as the dependent variable, we found that only birth weight emerged as independent determinant for this HRV parameter (Table 2). In the current study the birth weight explained 15% of the variance in RMSSD index (R^2^ = 0.15) (Table 2). On the other hand, multiple regression analysis revealed a significant interaction of the three independent variables birth weight, age and BMI in predicting PNN50 (Table 2). These independent variables explained 23% of the variability in PNN50 index (R^2^ = 0.23) (Table 2). Finally, regression analysis revealed that birth weight was the only significant predictor of HF, and explained 16% of the variance in HF (R^2^ = 0.16) (Table 2).

**Table 2:** Multiple Linear Regression Analyses for the Dependent Variables RMSSD, PNN50 and HF index.

	β	SE	CI (95%)	*P* Value
**RMSSD (ms)**				
Birth Weight	0.011	0.004	(0.002 to 0.019)	0.018
**PNN50 (%)**				
Birth Weight	0.008	0.003	(0.002 to 0.012)	0.007
Age	1.365	0.677	(0.019 to 2.710)	0.047
BMI	-0.753	0.308	(-1.364 to -0.142)	0.016
**HF (n.u.)**				
Birth Weight	0.007	0.003	(0.002 to 0.013)	0.013

Confounding factors (prematurity, birth weight, gender, age, BMI, physical activity index and SBP levels) were included in the same regression model. β: Parameter estimate indicating the alteration in HRV components caused by one unit of change in the independent variable; SE: Standard error; CI: Confidence interval; BMI—Body Mass Index; RMSSD—Root Mean Square of Successive Differences; pNN50—Percentage of Differences Between Adjacent Normal-to-Normal Intervals that is Greater than 50 Milliseconds; HF (n.u.)—High Frequency in Normalized Units.

## Discussion

It is known that the ANS development begins in fetal life and persists after birth [30, 31]. There is evidence suggesting that negative insults during intrauterine development promote functional disorders in the ANS, which may persist later in life [32]. Moreover, it should be noted that alterations in autonomic nervous function are associated with low birth weight and cardiometabolic abnormalities [17, 19, 20, 33]. Findings from the present study demonstrated that children with low birth weight have abnormal autonomic modulation, suggesting that variation in birth weight seems associated with the differences in autonomic function observed in later childhood. We found that the RMSSD, pNN50 index and HF band (n.u.) an indicative of the cardiac vagal modulation were positively correlated with birth weight. These findings preserved even after adjusting for prematurity, gender, age, BMI, physical activity index and SBP levels. One possible interpretation for our present data is that a vagal withdrawal, rather than a sympathetic overactivity, could precede the development of hypertension and other cardiometabolic diseases in children with low birth weight.

It has been reported that low birth weight is related to modulation of both the sympathetic and parasympathetic function from the prenatal period through childhood and adult life [17, 19, 20, 34–38]. Decreases in the HRV parameters were noted in newborns at term with low weight [34], while other studies observed a normal cardiac autonomic balance in these neonates [17, 35]. A recent study on children with low birth weight found a reduction in the overall activity of the ANS [15]. A study examining young adults with low birth weight found that these subjects possessed significantly increased parasympathetic parameters of the time domain [19]. Another study found increased sympathetic activation and reduced parasympathetic activity, demonstrating autonomic imbalance only in women with low birth weight who submitted to a stress test [20]. Ward et al. [36] also observed a negative correlation between birth weight and heart rate in response to psychological stress in women. Other studies found a significant correlation between low birth weight and increased sympathetic activity in adolescent twin pairs and young adults; however, this result did not observed gender differences [37, 38]. Our present findings indicate a reduction in activity of the parasympathetic ANS associated with low birth weight. This correlation remained significant even after controlling for multiple confounders. Therefore, our data are consistent and complements the results of previous studies reporting the presence of impairment in the vagal cardiac autonomic modulation in children born with low weight [15, 34, 39]. One important factor that could contribute to these discrepant findings is the use of birth weight as a unique marker of fetal development. The deleterious effects on development often vary in accordance to nature of the cause and period of exposure to adverse factors which have led to low birth weight.

It is well known that lower HRV parameters are associated with increased mortality due to heart failure, coronary heart disease and ischemic cardiomyopathy during adulthood [40]. There is also evidence that decreased parasympathetic activity is correlated with high blood pressure and obesity in childhood [41]. Thus, significant correlation between lower HRV parameters and low birth weight found in healthy children may have adverse prognostic effects and serve as an early sign of cardiometabolic diseases. The reason why low birth weight promotes alterations in cardiac ANS activity among health individuals is unknown. Considering that ANS is stable from intrauterine life through the postnatal period [42–44], an inadequate fetal environment can lead to negative changes in ANS development and functionality, thus promoting long-lasting consequences later in life. It has been demonstrated that children born to parents with socioeconomic problems have a higher probability of low birth weight and ANS dysfunction [45]. Interestingly, maternal prenatal stress could have negative effects on fetal ANS [46]. There is also evidence that prenatal supplementation with zinc has a positive effect on birth weight [47] and fetal HRV measures [48, 49]. Caulfield et al. [50] found that the beneficial effects of prenatal zinc supplementation on the ANS balance persisted until early infancy, suggesting that the nutritional status of the mother is important to fetal ANS development. These findings are consistent with the premise that modification in the fetal environment could alter ANS development. More detailed data about issues during pregnancy should be obtained in an attempt to identify specific periods during gestation that are important for the programming of the fetal autonomic system. Other potential mechanism that would explain the impairment of the vagal modulation observed in children with low birth weight could be mediated by renin-angiotensin system (RAS). There is evidence that angiotensin II inhibits parasympathetic modulation [51]. In accordance to this, an inverse correlation has been described between birth weight with both angiotensin II and ACE activity [52–54]. This is important since children with low birth weight with higher plasma ACE activity would presumably have high angiotensin II levels and lower associated levels of cardiac vagal activity. Therefore, it is possible that the RAS hyperactivity could, at least in part, mediate the impairment of cardiac vagal tone observed in low birth weight children. It is noteworthy, however, that complementary researches are necessary to determine the mechanisms involved as well as the potential influence of birth weight on the cardiac autonomic modulation.

The limitations of this study include the small sample size based on a cross-sectional analysis, which prevent us from establishing causal relationships. Despite these, our study was able to replicate findings reported in individuals from developed countries. In conclusion, our study revealed that children born with low weight presents detriment of the cardiac vagal autonomic modulation. Therefore, the high risk of cardiometabolic diseases among these children could be attributed, at least in part, to a reduction in the protective actions of the cardiac vagal tone. The interaction between low birth weight and reduction in parasympathetic regulation observed in the present study occurs independently of prematurity, gender, age, BMI, physical activity index and SBP levels.
